# Cannabis Use Is Associated With Lower COVID-19 Susceptibility but Poorer Survival

**DOI:** 10.3389/fpubh.2022.829715

**Published:** 2022-04-01

**Authors:** Da Huang, Roubing Xu, Rong Na

**Affiliations:** ^1^Ruijin Hospital, Shanghai Jiao Tong University School of Medicine, Shanghai, China; ^2^Department of Surgery, Queen Mary Hospital, The University of Hong Kong, Pok Fu Lam, Hong Kong SAR, China

**Keywords:** cannabis, COVID-19, survival, susceptibility, Comorbidity Index

## Abstract

**Objectives:**

To investigate the impact of cannabis use on the infection and survival outcomes of COVID-19.

**Study Design:**

Cross-sectional study based on the UK Biobank (UKB) dataset.

**Methods:**

We identified 13,099 individuals with cannabis smoking history in the UKB COVID-19 Serology Study. The Charlson-Quan Comorbidity Index was estimated using inpatient ICD-10 records. Multivariable logistic regression characterized features associated with COVID-19 infection. Cox models determined the hazard ratios (HR) for COVID-19-related survival.

**Results:**

Cannabis users were more likely to getting COVID-19 (odds ratio: 1.22, *P* = 0.001) but multivariable analysis showed that cannabis use was a protective factor of COVID-19 infection (adjusted odds ratio: 0.81, *P* = 0.001). Regular cannabis users, who smoked more than once per month, had a significantly poorer COVID-19-related survival, after adjusting for known risk factors including age, gender, smoking history, and comorbidity (adjusted hazard ratio: 2.81, *P* = 0.041).

**Conclusions:**

The frequency of cannabis use could be considered as a candidate predictor for mortality risk of COVID-19.

## Introduction

With nearly 120 million confirmed cases and 2 million deaths to date, the 2019 coronavirus disease (COVID-19) has become one of the most critical public health challenges in the twenty first century (1). Patients with COVID-19 would have heterogeneous severity, from mild-to-moderate cases with excellent survival to severe or critical cases with lethal outcomes (2). Risk factors of poorer survival were also reported, including age, gender, diabetes, respiratory disease, cancers, etc. (3). However, whether cannabis use would affect the infection and outcomes of COVID-19 is unknown.

Legislation of cannabis use has been approved for many years in most of the European and North American countries (4). In the United Kingdom, cannabis is classified as a Class B drug and is illegal for recreational use. However, individuals found with cannabis will usually be issued with a warning or an on-the-spot fine by police (5). As the most widely used addictive substance, its effect on health and disease outcomes has not been elucidated. Therefore, in the present study, we seek to investigate whether cannabis use and its frequency would be associated with COVID-19 susceptibility and related survival based on the UK Biobank (UKB) COVID-19 Serology Study, a prospective population cohort with available COVID-19 test results (6–8).

## Methods

A total of 13,099 individuals were recorded with cannabis smoking history (including ever/never use of cannabis and the maximum frequency of cannabis taken) via the additional follow-up assessment using an on-line questionnaire (last updated in July 2017) in the UKB COVID-19 subset (8, 9). Among them, we identified 1,925 patients (1,000 males, 869 females, and 56 missing) diagnosed with COVID-19 according to the COVID-19 test record (8) (at least one positive from May to December 2020) and death registration with International Classification of Diseases version 10 (ICD-10) code U071. We calculated the Charlson-Quan Comorbidity Index (CCI) based on inpatient ICD-10 records, by summing all the weights of each comorbidity category (10).

## Results

Univariable logistic regression analysis showed that individuals with cannabis smoking history were more likely to be diagnosed with COVID-19 [odds ratio (OR) 1.22, 95% confidence interval (95% CI) 1.09–1.37, *P* = 0.001; Table 1]. However, after adjusting for age, gender, race, smoking history, and CCI, the results indicated that cannabis use could significantly lower the risk of getting COVID-19 [adjusted OR (AOR) 0.81, 95% CI 0.71–0.92, *P* = 0.001]. Among individuals who have used multiple times of cannabis, for example, ≥1 time per month, the risk would be even lower (AOR 0.71, 95% CI 0.58–0.86, *P* < 0.001). After a median follow-up of 59 days among 1,925 COVID-19-positive patients, regular cannabis use (≥1 time per month) was found to be a significant risk factor for poorer COVID-19-related survival [adjusted hazard ratio (AHR) 2.81, 95% CI 1.04–7.59, *P* = 0.041; Table 2] in multivariable Cox regression analysis. No significant association was observed between cannabis use and overall survival among COVID-19 patients. We performed several subgroup analyses across age groups [stratified by the median age (70 years) of the entire cohort] and race. In Supplementary Table S1, the association between cannabis use and COVID-19 risk was still significant in elder subgroups (age ≥70 years). In Cox models, regular cannabis use (≥1 time per month) was still significantly associated with COVID-19-related survival in younger subgroup (AHR: 4.58, *P* = 0.048, Supplementary Table S2). The above results did not change the main conclusion of the present study. In subgroup analyses across race group, similar results were found in white groups (data not shown). The UKB cohort mainly included white population (>95%). The sample size of other races was so limited that no significant association was found.

**Table 1:** Logistic regression analyses on COVID-19 Infection in entire cohort (*N* = 13,099).

**Characteristics**		**Univariable analysis**		**Multivariable analysis**	
		**OR (95% CI)**	***P***	**AOR** **(95% CI)**	***P***
Age, years		0.93 (0.92–0.93)	<0.001	0.92 (0.92–0.93)	<0.001
Male (vs. female)		1.05 (0.95–1.15)	0.376	1.23 (1.11–1.36)	<0.001
White (vs. others)		0.73 (0.58–0.92)	0.007	0.96 (0.76–1.23)	0.780
Ever smoke (vs. never)		0.99 (0.90–1.09)	0.836	1.21 (1.09–1.35)	<0.001
Comorbidity Index^†^		0.91 (0.88–0.95)	<0.001	0.99 (0.95–1.03)	0.601
Ever taken cannabis (vs. others)	Any times	1.22 (1.09–1.37)	0.001	0.81 (0.71–0.92)	0.001
	≥3 times	1.24 (1.07–1.42)	0.003	0.76 (0.65–0.88)	<0.001
	≥1 /month^‡^	1.17 (0.98–1.39)	0.087	0.71 (0.58–0.86)	<0.001

*OR, odds ratio; 95% CI, 95% confidence interval; AOR, adjusted odds ratio.*

*^†^Charlson-Quan Comorbidity Index was calculated by summing all the weights (from 1 to 6) of each comorbidity category based on inpatient ICD-10 records.*

*^‡^Maximum frequency of taking cannabis*.

**Table 2:** Cox models in COVID-19 positive subset (*N* = 1,925).

**Characteristics**		**COVID-related survival**				**Overall survival**			
		**HR (95% CI)**	***P***	**AHR (95% CI)**	***P***	**HR (95% CI)**	***P***	**AHR (95% CI)**	***P***
Age, years		1.18 (1.13–1.24)	<0.001	1.17 (1.11–1.23)	<0.001	1.18 (1.13–1.23)	<0.001	1.17 (1.12–1.23)	<0.001
Male (vs. female)		2.96 (1.56–5.63)	0.001	1.79 (0.93–3.45)	0.082	2.40 (1.39–4.16)	0.002	1.48 (0.85–2.60)	0.168
White (vs. others)		2.40 (0.33–17.41)	0.385	0.89 (0.12–6.63)	0.908	1.69 (0.41–6.95)	0.465	0.68 (0.16–2.88)	0.601
Ever smoke (vs. never)		2.90 (1.56–5.39)	0.001	1.71 (0.88–3.34)	0.113	2.43 (1.42–4.15)	0.001	1.41 (0.79–2.50)	0.245
Comorbidity Index^†^		1.43 (1.28–1.59)	<0.001	1.25 (1.09–1.44)	0.001	1.41 (1.27–1.55)	<0.001	1.23 (1.08–1.39)	0.002
Ever taken cannabis (vs. others)	Any times	0.50 (0.23–1.12)	0.092	0.87 (0.38–2.02)	0.753	0.52 (0.26–1.05)	0.068	0.89 (0.42–1.86)	0.749
	≥3 times	0.69 (0.27–1.73)	0.426	1.58 (0.60–4.16)	0.357	0.54 (0.22–1.36)	0.191	1.17 (0.45–3.02)	0.750
	≥1/month^‡^	1.28 (0.51–3.24)	0.598	2.81 (1.04–7.59)	0.041	1.00 (0.40–2.49)	0.993	1.93 (0.73–5.08)	0.185

*HR, hazard ratio; 95% CI, 95% confidence interval; AHR, adjusted hazard ratio.*

*^†^Charlson-Quan Comorbidity Index was calculated by summing all the weights (from 1 to 6) of each comorbidity category based on inpatient ICD-10 records.*

*^‡^Maximum frequency of taking cannabis*.

## Discussion

In this mid-aged/elderly cohort (median age: 70 years), individuals with younger age were more likely to be infected by COVID-19 in our study, which was similar as reported studies (11); meanwhile, cannabis consumption was significantly popular in younger people (12). The age might be considered as a major confounder in this analysis. Therefore, different results were observed between univariable and multivariable analyses. Our study suggested that cannabis use would be a protective factor of COVID-19 infection. Several studies suggested self-isolated individuals during the COVID-19 pandemic might increase cannabis use (13, 14), indicating that self-isolation could be the confounder between cannabis and COVID-19. However, based on the reported studies investigating the behavior change in cannabis users, individuals using cannabis might be easily satisfied by the substance use and show a reduction of social activities (12). Therefore, we believed that cannabis use was a direct protective factor for COVID-19 infection.

COVID-19 patients with complex health conditions were reported to have significantly poorer survival (2, 3). In the present study, we evaluated the complex health conditions using the CCI. We observed similar results that the CCI was significantly associated with COVID-19-related survival (HR 1.43, *P* = 0.001) and overall survival (HR 1.41, *P* = 0.001). Here, after adjusting for multiple variables including the CCI, cannabis use was considered a very significant risk factor for poor COVID-19-related survival but not for overall survival. The underneath biological mechanisms were still unclear and worth further investigation. Previous studies suggested that cannabis use was associated with cardiovascular and respiratory disorders (12). Health conditions might be more fragile in people with cannabis smoking history than others. They might be more likely to turn into a critical condition after COVID-19 infection. In addition, cannabis users are often comorbid with poor sleep behavior, which has been also shown to predict COVID-19 mortality (15).

There were several limitations of this study. First, a relatively small number of COVID-19 patients with cannabis smoking history information were found in UKB, which was the major limitation. However, to our knowledge, this is the largest cohort investigating the association between cannabis use and COVID-19 to date. Second, the individuals were enrolled between May and December 2020 in the UKB COVID-19 Serology Study (8). The timing was during the original wave and start of the alpha variant but not the latest variants (such as delta or omicron), when the mass vaccination was also not started. Third, the cannabis usage data was self-reported and last updated in 2017, which predated the serology study by 3 years. The results could be updated with the additional release from the UKB in the future.

## Data Availability Statement

Publicly available datasets were analyzed in this study. This data can be found here: all the data were extracted from the UK Biobank. All data used in this research are publicly available to qualified researchers on application to the UK Biobank (www.ukbiobank.ac.uk).

## Author Contributions

RN conceived and designed the study. DH, RX, and RN extracted and analyzed the data. DH and RN wrote the manuscript. All authors contributed to the article and approved the final version.

## Conflict of Interest

The authors declare that the research was conducted in the absence of any commercial or financial relationships that could be construed as a potential conflict of interest.

## Publisher's Note

All claims expressed in this article are solely those of the authors and do not necessarily represent those of their affiliated organizations, or those of the publisher, the editors and the reviewers. Any product that may be evaluated in this article, or claim that may be made by its manufacturer, is not guaranteed or endorsed by the publisher.
