# Supplementary material for: Cannabis Use Is Associated With Lower COVID-19 Susceptibility but Poorer Survival
Source: Front Public Health. 2022 Apr 1;10:829715. doi: 10.3389/fpubh.2022.829715 (PMC9012397; doi:10.3389/fpubh.2022.829715)
Supplement: Supplementary file 1 [file Table_1.PDF]

Supplementary Material

Supplementary Table 1. Logistic regression analyses on COVID-19 Infection across different age groups.

Characteristics		Age ≥ 70 yrs (n = 6,777)				Age < 70 yrs (n = 6,350)			
		OR (95% CI)	*P*	AOR (95% CI)	*P*	OR (95% CI)	*P*	AOR (95% CI)	*P*
Age, years		0.93 (0.92-0.95)	<0.001	0.93 (0.92-0.94)	<0.001	0.95 (0.93-0.98)	<0.001	0.95 (0.92-0.97)	<0.001
Male (vs. Female)		1.13 (1.00-1.28)	0.047	1.20 (1.06-1.36)	0.005	1.30 (1.09-1.54)	0.004	1.28 (1.07-1.53)	0.006
White (vs. Others)		0.91 (0.71-1.18)	0.490	0.97 (0.74-1.26)	0.801	1.00 (0.53-1.86)	0.993	0.99 (0.52-1.92)	0.988
Ever Smoke (vs. Never)		1.00 (0.88-1.12)	0.943	1.15 (1.00-1.32)	0.043	1.34 (1.13-1.60)	0.001	1.34 (1.12-1.61)	0.001
Comorbidity Index†		0.92 (0.87-0.97)	0.002	0.95 (0.90-1.01)	0.076	1.04 (0.98-1.10)	0.184	1.03 (0.98-1.09)	0.266
Ever Taken Cannabis (vs. Others)	Any times	0.93 (0.81-1.06)	0.268	0.81 (0.70-0.94)	0.005	1.03 (0.80-1.32)	0.832	0.86 (0.66-1.12)	0.260
	≥3 times	0.92 (0.79-1.08)	0.319	0.77 (0.65-0.92)	0.004	0.93 (0.64-1.35)	0.710	0.75 (0.51-1.11)	0.152
	≥1 /month‡	0.87 (0.71-1.05)	0.150	0.71 (0.58-0.88)	0.002	0.97 (0.61-1.55)	0.907	0.78 (0.49-1.26)	0.315

Abbreviation: OR, odds ratio; 95% CI, 95% confidence interval; AOR, adjusted odds ratio.

† Charlson-Quan Comorbidity Index was calculated by summing all the weights (from 1 to 6) of each comorbidity category based on inpatient ICD-10 records.

‡ Maximum frequency of taking cannabis.

Supplementary Table 2. Cox models on Covid-related survival across different age groups in COVID-19 positive subset (n = 1,925).

Characteristics		Age ≥ 70 yrs (n = 570)				Age < 70 yrs (n = 1,355)			
		HR (95% CI)	*P*	AHR (95% CI)	*P*	HR (95% CI)	*P*	AHR (95% CI)	*P*
Age, years		1.16 (1.05-1.28)	0.003	1.15 (1.03-1.27)	0.010	1.19 (1.03-1.38)	0.017	1.22 (1.04-1.45)	0.018
Male (vs. Female)		2.44 (1.15-5.16)	0.020	1.90 (0.89-4.09)	0.099	1.71 (0.46-6.36)	0.425	1.47 (0.39-5.60)	0.569
White (vs. Others)		not applicable	/	not applicable	/	0.56 (0.07-4.45)	0.587	0.38 (0.05-3.23)	0.378
Ever Smoke (vs. Never)		2.14 (1.04-4.38)	0.038	1.58 (0.75-3.32)	0.229	2.08 (0.59-7.37)	0.256	2.17 (0.49-9.54)	0.304
Comorbidity Index†		1.32 (1.16-1.51)	<0.001	1.25 (1.07-1.46)	0.004	1.44 (1.13-1.82)	0.003	1.25 (0.90-1.75)	0.189
Ever Taken Cannabis (vs. Others)	Any times	0.71 (0.25-2.00)	0.516	0.78 (0.27-2.26)	0.653	1.04 (0.27-4.01)	0.958	0.96 (0.22-4.21)	0.956
	≥3 times	0.96 (0.23-3.99)	0.956	1.20 (0.29-5.05)	0.800	2.00 (0.52-7.72)	0.317	2.25 (0.50-10.11)	0.289
	≥1 /month‡	1.73 (0.42-7.17)	0.453	1.86 (0.43-8.12)	0.406	3.69 (0.96-14.29)	0.058	4.58 (1.01-20.78)	0.048

Abbreviation: HR, hazard ratio; 95% CI, 95% confidence interval; AHR, adjusted hazard ratio.

† Charlson-Quan Comorbidity Index was calculated by summing all the weights (from 1 to 6) of each comorbidity category based on inpatient ICD-10 records.

‡ Maximum frequency of taking cannabis.
